# Pulmonary inhalation-perfusion scintigraphy in the evaluation of bronchoscopic treatment of bronchopleural fistula

**DOI:** 10.1590/0100-3984.2017.0133

**Published:** 2018

**Authors:** Carla Rachel Ono, Miguel Lia Tedde, Paulo Rogerio Scordamaglio, Carlos Alberto Buchpiguel

**Affiliations:** 1 Nuclear Medicine Division, Instituto de Radiologia do Hospital das Clínicas da Faculdade de Medicina da Universidade de São Paulo (InRad/HC-FMUSP), São Paulo, SP, Brazil.; 2 Department of Thoracic Surgery, Instituto do Coração do Hospital das Clínicas da Faculdade de Medicina da Universidade de São Paulo (InCor/HC-FMUSP), São Paulo, SP, Brazil.; 3 Respiratory Endoscopy Division, Instituto do Coração do Hospital das Clínicas da Faculdade de Medicina da Universidade de São Paulo (InCor/HC-FMUSP), São Paulo, SP, Brazil.

**Keywords:** Radionuclide imaging/methods, Radioactive tracers, Bronchial fistula, Septal occluder device, Lung

## Abstract

**Objective:**

To evaluate the use of pulmonary inhalation-perfusion scintigraphy as an alternative method of investigation and follow-up in patients with bronchopleural fistula (BPF).

**Materials and Methods:**

Nine patients with BPFs were treated through the off-label use of a transcatheter atrial septal defect occluder, placed endoscopically, and were followed with pulmonary inhalation-perfusion scintigraphy, involving inhalation, via a nebulizer, of 900-1300 MBq (25-35 mCi) of technetium-99m-labeled diethylenetriaminepentaacetic acid and single-photon emission computed tomography with a dual-head gamma camera.

**Results:**

In two cases, there was a residual air leak that was not identified by bronchoscopy or the methylene blue test but was detected only by pulmonary inhalation-perfusion scintigraphy. Those results correlated with the evolution of the patients, both of whom showed late signs of air leak, which confirmed the scintigraphy findings. In the patients with complete resolution of symptoms and fistula closure seen on bronchoscopy, the scintigraphy was completely negative. In cases of failure to close the BPF, the scintigraphy confirmed the persistence of the air leak. In two patients, scintigraphy was the only method to show residual BPF, the fistula no longer being seen on bronchoscopy.

**Conclusion:**

We found pulmonary inhalation-perfusion scintigraphy to be a useful tool for identifying a residual BPF, as well as being an alternative method of investigating BPFs and of monitoring the affected patients.

## INTRODUCTION

A bronchopleural fistula (BPF) has been defined as a direct communication between a bronchus and the pleural space. A peripheral BPF is a fistulous communication between the airway distal to the segmental bronchi or lung parenchyma and the pleura, occurring after infection, rheumatic diseases, necrotizing pneumonia, empyema, radiotherapy, bulla rupture, or interventional procedures. A central BPF is a fistulous communication between the trachea or segmental bronchi and the pleura, occurring after lung resection or traumatic disruption of the tracheobronchial tree. Although rare, a BPF is one of the most serious life-threatening complications of pulmonary resection^(^^1^^,^^2^^)^.

Dehiscence of the bronchial stump after pulmonary resection continues to be the most common cause of BPF. The reported incidence of BPF ranges from 0.5% to 3.0% after lobectomy and from 2% to 20% after pneumonectomy, its occurrence typically being associated with high morbidity and mortality. In patients with BPF, the air leak through the fistula makes the situation more dramatic because it impairs ventilation and phonation, as well as increasing pleural space secretions^(^^3^^,^^4^^)^.

Bronchoscopy is one of the most accurate methods to identify a BPF, which is often challenging, especially when the BPF is small^(^^5^^)^.

A crucial step in the treatment of patients with BPF involves drainage of the pleural space, which, by definition, is contaminated, the drainage protecting the contralateral lung from leakage of pleural fluid via the BPF path. When possible, early central BPFs should be treated surgically, through repair of the bronchial stump. Although surgical correction is the treatment of choice, some patients are not suitable candidates for another surgical resection. In this scenario, a variety of minimally invasive transbronchial methods, including the use of occlusive agents (e.g., fibrin sealants), coils, stents, or one-way valves, have been employed in order to close the central BPF directly^(^^6^^-^^10^^)^. Such bronchoscopic treatments are successful only when the BPF is small. For larger fistulas, such as those caused by complete dehiscence of the stump (the so-called "total" fistulas), none of the endoscopic treatments have proven effective.

In the present study, we opted to treat patients with total BPF through bronchoscopic placement of occluders originally developed for percutaneous closure of cardiac septal defects^(^^11^^)^. After the treatment, we followed those patients by performing periodic bronchoscopic evaluations. However, the presence of the occluder within the bronchial stump decreases the accuracy of such evaluations.

The purpose of this study was to evaluate the value and usefulness of nuclear pulmonary inhalation-perfusion scintigraphy as an alternative method of investigation and follow-up of patients with BPFs.

## MATERIALS AND METHODS

### Study design and patient sample

**Figure 1:**
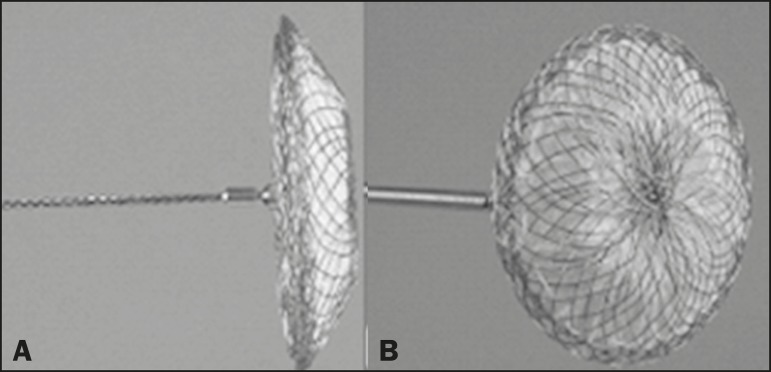
The Occlutech Figulla ASD N device. **A:** Lateral view. **B:** Frontal view.

This was a prospective study evaluating the safety and efficacy of endoscopic treatment of total BPFs through the off-label use of a transcatheter atrial septal defect occluder (Figulla ASD N; International Occlutech AB, Helsingborg, Sweden), as depicted in Figure 1, and the use of a pulmonary inhalation-perfusion scintigraphy as a method of detecting a residual BPF. The study was approved by the Committee for the Analysis of Research Projects of the Hospital das Clínicas da Faculdade de Medicina da Universidade de São Paulo (HC-FMUSP), reference no. 1089/09, and was registered with ClinicalTrials.gov (identifier: NCT01153074; http://www.clinicaltrials.gov/).

The study design included an initial bronchoscopic examination to measure the BPF diameter, assess the degree of mucosal inflammation along the fistula trajectory, and obtain biopsy samples to exclude the presence of residual disease. Inhalation-perfusion scintigraphy was performed at baseline (before the occluder was put in place). Patients were followed through periodic bronchoscopy. A final evaluation, conducted at 12 months after the bronchoscopic occlusion of the fistulas, included a complete clinical interview (to check for residual clinical symptoms such as dyspnea and difficulty in phonation) and a check for air leaks through the thoracostomy or chest tube, as well as bronchoscopy with a methylene blue test and another inhalation-perfusion scintigraphy examination.

The study sample comprised nine patients with total BPFs, all of whom had undergone open drainage of the pleural space by pleurotomy or chest tube and were not suitable candidates for surgical correction. The demographic, anthropometric, and clinical characteristics of the patients, including the Medical Research Council dyspnea score^(^^12^^)^, are shown in Table 1.

**Table 1:** Demographic and clinical characteristics of the patients.

Patient	Age (years)	Gender	Weight (kg)	Height (m)	BMI (kg/m^2^)	MRC score	Difficulty in phonation
1	45	Male	62	1.64	23.1	4	Yes
2	43	Male	45	1.74	14.9	5	Yes
3	42	Male	48	1.66	17.4	5	Yes
4	55	Male	74	1.74	24.5	5	Yes
5	58	Female	41	1.74	17.7	5	Yes
6	45	Male	49	1.74	19.9	5	Yes
7	72	Male	48	1.74	18.7	5	Yes
8	38	Female	48	1.74	20.0	4	No
9	30	Male	71	1.74	23.2	5	Yes

BMI, body mass index; MRC, Medical Research Council (dyspnea scale).

The underlying disease, the type of surgery performed, the location of the BPF, the treatment of the ipsilateral pleural space, the time since the initial appearance of the fistula, and the number of previous (surgical or bronchoscopic) attempts at closure of the fistula are described in Table 2.

**Table 2:** The underlying disease, type of surgery performed, characteristics of the BPF, pleural space treatment, time since the occurrence of the fistula, and previous (surgical or bronchoscopic) attempts at closure.

				Pleural space	Duration of	Previous closure
Patient	Underlying disease	Type of surgery	BPF location	treatment	fistula (years)	attempts (n)
1	Tuberculosis and fungal infection	Left pneumonectomy	Left main bronchus	Pleurotomy	1	2
2	Tuberculosis	Upper left lobectomy +	Upper left bronchus +	Pleurotomy	1	2
		complete pneumonectomy	lower left bronchus			
3	Tuberculosis	Upper right lobectomy +	Intermediate bronchus	Pleurotomy	16	4*
		complete pneumonectomy				
4	Squamous cell carcinoma	Right pneumonectomy	Right main bronchus	Chest tube (open	0.25	0
				drainage)		
5	Tuberculosis	Right pneumonectomy	Right main bronchus	Pleurotomy	0.25	0
6	Tuberculosis	Right pneumonectomy	Right main bronchus	Pleurotomy	8	11*
7	Squamous cell carcinoma	Left pneumonectomy	Left main bronchus	Pleurotomy	17	3
8	Necrotizing pneumonia (kidney	Upper right lobectomy	Upper right bronchus	Chest tube	0.33	3*
	transplant recipient)			(closed drainage)		
9	Tuberculosis	Right pneumonectomy	Right main bronchus	Pleurotomy	11	3*

* Surgical.

The median time from the BPF occurrence to treatment was 5.93 years (range, 0.25-17.0 years), and there had been previous (surgical or bronchoscopic) attempts at closure of the fistula in all of patients except patients 3 and 4. After the first bronchoscopic evaluation, the patients were submitted to a baseline pulmonary inhalation-perfusion scintigraphy. The inclusion criterion was having a BPF detected by scintigraphy. The technique utilized to place the occluders has been previously described in detail^(^^13^^)^.

### Pulmonary inhalation-perfusion scintigraphy

All scintigraphy procedures were performed in the Nuclear Medicine Division of the HC-FMUSP Department of Radiology, using a dual-head gamma camera (E-Cam; Siemens Medical Solutions, Chicago, IL, USA). 

Prior to inhalation of the radiopharmaceutical and the acquisition of images, the thoracostomy was occluded with a bandage or the chest tube was closed. Each patient inhaled, via a nebulizer, 900-1300 MBq (25-35 mCi) of technetium-99m-labeled diethylenetriaminepentaacetic acid (^99m^Tc-DTPA). All patients were kept in the upright position, and, during tidal breathing with the nose occluded, the aerosolized ^99m^Tc-DTPA was administered through a mouthpiece over a period of five minutes. The estimated level of activity that was reached and maintained in the lungs was 20-40 MBq (0.5-1.0 mCi).

### Image acquisition

After the inhalation of the radiopharmaceutical, planar images of the chest were obtained in anterior, posterior, lateral, and oblique views. With a 128 × 128 matrix and a low-energy, high-resolution collimator, each planar image accumulated 500 K counts. The system was calibrated for an energy photopeak of 140 keV with a 15% window. One additional image was acquired in the anterior view of the chest with a flood source behind the patient in order to delineate the contours of the body.

### Perfusion scintigraphy

The perfusion scintigraphy scans were obtained after the inhalation scintigraphy scans. The perfusion scans were acquired after intravenous administration of 185 MBq (5 mCi) of ^99m^Tc-labeled macroaggregated albumin (^99m^Tc-MAA).

### Imaging acquisition

Planar images of the chest were obtained in anterior, posterior, lateral, and oblique views. With a 128 × 128 matrix and a low-energy, high-resolution collimator, each planar image accumulated 1500 K counts. The system was calibrated for an energy photopeak of 140 keV with a 15% window.

### Follow-up and statistical analysis

**Figure 2:**
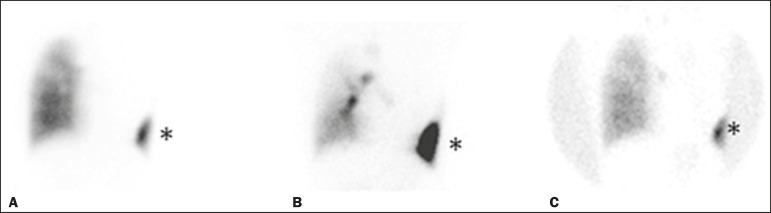
Patient submitted to left pneumonectomy and inhalation-perfusion scintigraphy. **A:** Scintigraphy with ^99m^Tc-MAA inhalation, anterior view in the perfusion scan. **B:** Scintigraphy with ^99m^Tc-DTPA inhalation, anterior view in the ventilation scan. **C:** Scintigraphy with ^99m^Tc-DTPA inhalation, anterior view and a silhouette contour in the inhalation scan. The images demonstrated no radiopharmaceutical activity in the left lung. Note the intense radiopharmaceutical activity at the bandage occluding the wound (asterisk) on the left side of the chest wall, demonstrating the BPF, in the inhalation scan. In the perfusion scan (**A**), residual radiopharmaceutical activity from the inhalation scan can be seen.

**Figure 3:**
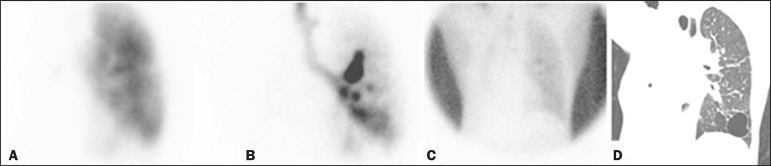
Patient submitted to right pneumonectomy and inhalation-perfusion scintigraphy. **A:** Scintigraphy with ^99m^Tc-MAA inhalation, anterior view in the perfusion scan. **B:** Scintigraphy with ^99m^Tc-DTPA inhalation, anterior view in the ventilation scan. **C:** Scintigraphy with ^99m^Tc-DTPA inhalation, anterior view and a silhouette contour in the ventilation scan. **D:** Coronal computed tomography scan, with a lung window setting. The images demonstrated no radiopharmaceutical activity in the right lung. There were no signs of BPF.

We have provided two examples of scintigraphy examinations. One shows the ^99m^Tc-DTPA activity in the bandage occluding the wound on the left side of the chest wall, revealing the BPF (Figure 2). The other example demonstrates no signs of a BPF in a patient submitted to right pneumonectomy (Figure 3).

**Figure 4:**
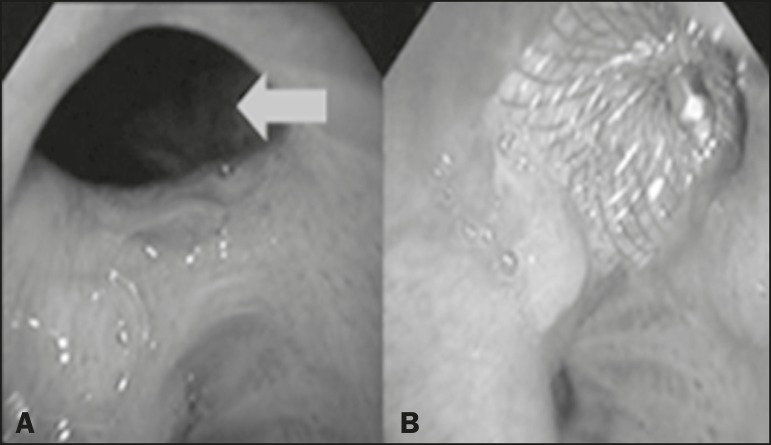
Bronchoscopic view of a BPF in the upper right bronchus: **A:** Pretreatment. **B:** At 12 months after placement of the occluder in the fistula trajectory.

As previously mentioned, bronchoscopy was repeated at 12 months after placement of the occluder, in order to evaluate the effectiveness of the treatment (Figure 4). The possibility of residual fistula was explored through instillation of methylene blue into the treated bronchial stump. The visualization of the dye in the pleural drain or pleurotomy was considered a strong indicator of residual air leak. Another ventilation scan was also acquired at that time.

A favorable outcome was defined as complete closure of the fistula, as confirmed by bronchoscopy, together with a negative methylene blue test or significant improvement of symptoms and the device being well positioned in the stump. 

Continuous variables are expressed as means and standard deviations, whereas categorical variables are expressed as absolute and relative frequencies.

## RESULTS

Most (66.7%) of the BPFs evaluated were on the right side, and the mean diameter was 15 ± 3.65 mm (range, 6-17 mm). The initial bronchoscopic evaluation showed that all fistulas presented well-defined edges, suggesting a chronic process, and the biopsy of the mucosa along the fistula trajectory excluded active local disease in all cases. The pulmonary inhalation-perfusion scintigraphy obtained at baseline confirmed the presence of the radioactive tracers in the thoracostomy bandage in all nine patients (Figure 2).

In two patients (cases 2 and 4) the occluder was withdrawn: in one, because a wire broke during an attempt to reposition the device; in the other, because the patient was severely undernourished and had not developed granulation tissue after one year. Two other patients (corresponding to cases 5 and 6) died from unrelated causes, which precluded their inclusion in the final evaluation. Therefore, the final sample comprised five patients. The results of the baseline pulmonary inhalation-perfusion scintigraphy, together with aspects observed at 12 months after placement of the occluder, including the clinical symptoms, the bronchoscopic aspect of the bronchial stump, the methylene blue test results, and the results of the final scintigraphy are presented in Table 3.

**Table 3:** Baseline inhalation-perfusion scintigraphy, together with aspects observed at 12 months after placement of the occluder, including clinical symptoms, as well as the results of the bronchoscopic evaluation of the bronchial stump, methylene blue test, and final scintigraphy.

	Baseline		After occluder placement		
		Clinical		Methylene	Final
Patient	scintigraphy	symptoms	Bronchoscopy	blue test	scintigraphy
1	Positive	Absent	Closed	Negative	Negative
2	Positive	Present	Device withdrawn	Positive	Positive
3	Positive	Improved	Closed	Negative	Positive
4	Positive	Present	Device withdrawn	Positive	Positive
5	Positive	*			
6	Positive	*			
7	Positive	Absent	Closed	Negative	Negative
8	Positive	Absent	Closed	Negative	Negative
9	Positive	Improved	Closed	Negative	Positive

* Died of unrelated causes.

The placement of the device in the bronchial stump promoted a significant improvement of clinical symptoms in the majority of the patients. In most cases, the patients showed improved respiration and phonation, as well as a reduction in pleural secretions and an improvement in their nutritional status. The improvement in clinical symptoms persisted while the device remained in the stump.

In two cases, there was a residual air leak that was not identified by bronchoscopy or the methylene blue test but was detected only by pulmonary inhalation-perfusion scintigraphy. Those results correlated with the evolution of the patients, both of whom showed late signs of air leak, confirming the scintigraphy findings.

## DISCUSSION

A BPF and the subsequent pleural space contamination constitute one of the most serious postoperative complications after pulmonary resection. If not drained, the massive secretions from the pleural space can be aspirated through the fistula, choking the patient or contaminating the contralateral lung. That is why all of the patients in our sample had a chest tube or a pleurotomy. However, drainage of the pleural space creates a route for a major air leak that can hamper respiration and phonation.

The rational for bronchoscopic treatment of BPF in patients whose clinical condition precludes surgical correction is the fact that the placement of an occluder in the bronchial stump results in rapid improvement of symptoms. In a previous study conducted by our group, we reported our experience with the bronchoscopic treatment of fistulas^(^^14^^-^^16^^)^.

A BPF can be detected by several imaging modalities other than bronchoscopy, including chest X-ray and multidetector computed tomography employing advanced image post-processing techniques. However, the presence of the occluder within the bronchial stump, in the BPF trajectory, could produce an artifact in the above mentioned radiographic methods and could preclude better evaluation through bronchoscopy. That is the rationale for choosing lung inhalation scintigraphy as an alternative imaging method to evaluate the BPF before and after endoscopic treatment.

Greyson et al.^(^^17^^)^ were the first to demonstrate that scintigraphy with inhalation of a radionuclide (^99m^Tc-albumin colloid fog) is a simple and accurate test for the detection of a BPF.

In addition to ^99m^Tc-albumin colloid fog, a variety of radioactive tracers, including ^99m^Tc-sulfur colloid, ^99m^Tc-DTPA, or a gas like ^133^Xe, could be alternative radiopharmaceuticals for use in inhalation-perfusion scintigraphy^(^^18^^,^^19^^)^.

Mark et al.^(^^20^^)^ reported their experience using inhalation-perfusion scintigraphy with ^99m^Tc-DTPA inhalation in 28 patients who had undergone pneumonectomy, showing that, for the detection of a BPF, the method had a sensitivity of 78%, a specificity of 100%, and an accuracy of 86%. Nevertheless, it lacks accuracy in the detection of very small BPFs, is time-consuming, and requires patient cooperation, which can be difficult during the postoperative period, when the patients could be on mechanical ventilation, could be critically ill, or could have sepsis.

Pulmonary inhalation-perfusion scintigraphy has other known limitations. The turbulent flow in the tracheobronchial tree, which promotes aerosol deposition, can lead to false-positive results in patients with chronic obstructive pulmonary disease. Therefore, this modality is currently used only when conventional bronchoscopy, virtual bronchoscopy, and multidetector computed tomography have all failed to identify a clinically suspected BPF^(^^21^^)^.

Although there is no standardization of pulmonary inhalation-perfusion scintigraphy for monitoring these cases, the technique used in the present study provided excellent results that were strongly correlated with clinical improvement and bronchoscopic findings, even in long-term clinical follow-up.

In the patients with complete resolution of symptoms, closure of the fistula confirmed by bronchoscopy, and no evidence of dye leakage, the inhalation-perfusion scintigraphy was completely negative. In cases of failure to close the BPF, the scintigraphy confirmed the persistence of the air leak.

In two (40%) of the five patients in our sample, scintigraphy was the only method to show residual BPF, which was not detected in the bronchoscopic assessment or in the methylene blue test (no dye extravasation into the pleural space). Therefore, to avoid events related to the severe sepsis that could occur if the space closed prematurely, the thoracostomy was maintained, thus minimizing the risk, in those two patients.

In the literature, ^99m^Tc-DTPA aerosol inhalation scintigraphy has been reported to have poor sensitivity. However, in the present study, the initial inhalation-perfusion scintigraphy detected BPF in all of the patients evaluated. That is probably because open drainage of the hemithorax (with thoracostomy or a chest tube) was employed in all of the patients in our sample. Such drainage likely promotes the flow directly out of the thoracic cavity, as well as allowing the detection of radioactive activity in the occlusive bandage or adjacent to the chest wall (on its outer face). The detection of radiopharmaceutical activity in the bandage or in the additional image obtained with the flood source facilitated the localization of such activity outside of the body^(^^22^^)^.

Our results shows that pulmonary inhalation-perfusion scintigraphy is a useful tool to identify residual BPFs, even when classical methods such as bronchoscopy and the blue methylene test fail to detect it. Additional studies with larger patient samples are needed in order to confirm our preliminary findings.
